# Altered Error Monitoring and Decreased Flanker Task Accuracy in Pediatric Obsessive-Compulsive Disorder

**DOI:** 10.21203/rs.3.rs-3943784/v1

**Published:** 2024-02-13

**Authors:** Gregory Hanna, Yanni Liu, Lauren Rentschler, Barbara Hanna, Paul Arnold, William Gehring

**Affiliations:** University of Michigan-Ann Arbor; University of Michigan-Ann Arbor; University of Michigan-Ann Arbor; University of Michigan-Ann Arbor; University of Calgary; University of Michigan-Ann Arbor

**Keywords:** Error Positivity, Error-Related Negativity, Flanker Task, Obsessive-Compulsive Disorder, Tic Disorder, Youth

## Abstract

The error-related negativity (ERN) and error positivity (Pe) are components of the event-related potential following an error that are potential mechanistic biomarkers of obsessive-compulsive disorder (OCD). The study examined the ERN, Pe, flanker task accuracy, and clinical measures in 105 OCD cases and 105 matched healthy controls (HC), ages 8 to 18 years, with 21 cases having a tic disorder history. Higher flanker task accuracy in all participants was associated with an increased ERN amplitude and increased difference between Pe and correct positivity amplitudes (ΔPe). Compared to HC, OCD cases had an increased ERN but *decreased* flanker task accuracy and ΔPe. Those differences were also significant in tic-related and non-tic-related OCD cases compared to HC. A lower ΔPe was associated in OCD cases with an earlier age at OCD symptom onset. The results support the hypothesis that OCD involves defects in an error monitoring system and suggest a reduced ΔPe may compromise error signaling and cause uncertainty about the correctness of a response.

## Introduction

Obsessive-compulsive disorder (OCD) is a heterogeneous and often chronic psychiatric disorder, with lifetime prevalence rates ranging from 1–3% [1]. OCD has a median age at onset of about 19 years, with about 25% of cases starting by 14 years [2]. OCD involves recurrent intrusive thoughts and repetitive behaviors or mental acts that vary in their content and are often associated with other psychiatric disorders [1]. About 22% of pediatric OCD cases have a comorbid tic disorder [3], with tic-related OCD (TR OCD) having an earlier age at onset than non-tic-related OCD (NTR OCD) [4].

Research on mechanistic biomarkers that precede or develop concurrently with onset of pediatric OCD may clarify its pathogenesis, improve diagnostic and preventive strategies, and provide treatment targets [5]. OCD has been hypothesized to involve persistent errors signals that cannot be eliminated by behavioral output [6]. In the context of cognitive control, performance monitoring refers to neural processes that support the continuous monitoring of thoughts and actions [7, 8]. Two components of the event-related potential (ERP) involved in performance monitoring are the error-related negativity (ERN or Ne), a negative deflection in the response-locked ERP waveform that peaks within 100 ms after error commission and is maximal at frontocentral electrodes, and the error positivity (Pe), a positive deflection in the response-locked ERP waveform that peaks between 200 and 500 ms after an erroneous response and is maximal at centroparietal electrodes [7–11]. The correct response negativity (CRN) and correct positivity (Pc) that occur after a correct response in the same time windows as the ERN and Pe, respectively, are distinguished from their counterparts by having lower amplitudes [7–11].

The ERN has been described as a neural marker of error monitoring processes [7–10], reinforcement learning [12], error-related distress [13], and the motivational significance of errors [14]. Thus, it is a unit of analysis in three domains of the Research Domain Criteria (RDoC) project: cognitive systems (cognitive control: performance monitoring), negative valence systems (sustained threat), and positive valence systems (reward learning) [15]. The ERN increases in magnitude throughout childhood and adolescence, indicating a prolonged maturation of the system underlying performance monitoring that may allow the ERN to adjust to defects in that system over time [6, 16].

Increased ERN amplitudes have been found in studies of adults and children with OCD using choice reaction time tasks eliciting response conflict, suggesting the ERN may serve as a biomarker for OCD [7, 8, 15, 17–19]. Studies with an enlarged ERN in adults with OCD have noted either normal or increased accuracy on response conflict tasks relative to healthy controls (HC) [7, 8, 15, 17–19]. However, our studies of older children and adolescents have shown increased ERN amplitudes but *decreased* flanker task accuracy in youth with OCD compared to HC [20, 21]. In contrast, we found augmented ERN amplitudes but normal flanker task accuracy in youth with anxiety disorders compared to HC [22] and no differences between youth with major depressive disorder and HC in either ERN amplitudes or flanker task accuracy [21].

The inconsistency between an enlarged ERN and decreased task accuracy suggests an altered Pe may compromise performance monitoring in pediatric OCD [11]. The Pe has been posited to reflect the post-decisional evidence accumulation process that is sensitive to decision accuracy, decision confidence, and subsequent adaptation in behavior [11]. It has a highly robust association with error detection [23] that varies with the level of confidence that an error has been made, with a higher Pe amplitude reflecting greater certainty [24]. A meta-analysis found no significant association between age and the Pe, except for a group comparison between younger and older adolescents, suggesting an alteration in the Pe may arise at or before the onset of OCD symptoms and correlate with symptom onset age [25].

One study found significant associations between higher ERN and Pe amplitudes and parent-reported obsessive-compulsive behaviors in a non-clinical sample of 10-year-old children [26]. However, no studies have examined the Pe and Pc in youth with OCD [8]. Of the eight studies examining the Pe in adults with OCD, one found a diminished Pe with the others finding no differences between OCD cases and HC [8, 27].

The following study was done with 105 older children and adolescents with a lifetime diagnosis of OCD and 105 age- and sex-matched healthy controls (HC) using a flanker task [20–22]. The first aim was to compare ERN and Pe measures and flanker task accuracy in OCD cases and HC. The second aim was to compare those measures in TR OCD cases, NTR OCD cases, and HC, because previous studies lacked adequate statistical power to assess them in the two OCD subtypes [8, 20, 28]. Since the largest group differences in the brain potentials were found with the ERN and ΔPe, those measures were used in subsequent analyses. The third aim was to do a multiple regression analysis in all participants to examine the association of flanker task accuracy with age, ERN, and ΔPe. The fourth aim was to do separate multiple regression analyses in all participants to examine the association of the ERN and ΔPe with age, flanker task accuracy, lifetime diagnosis of OCD, and Child Behavior Checklist/6–18 (CBCL/6–18) *DSM*-Oriented Scale scores and determine whether a dimensional measure of clinical symptoms may have a stronger association with either brain potential than a lifetime OCD diagnosis [29, 30]. The fifth aim was to do a multiple regression analysis in the OCD cases to examine the association of age at OCD symptom onset with age, lifetime diagnosis of tic disorder, ERN, and ΔPe. Because the ERN and Pe have different developmental trajectories, it was hypothesized the ΔPe may be more strongly associated than the ERN with age at OCD symptom onset (16, 25).

## Method

### Participants

Patients with OCD were recruited from the Department of Psychiatry at the University of Michigan and surrounding community. HC were recruited from the surrounding community and were matched to patients by age and sex. Participants were recruited using flyers and UM Health Research Studies (http://www.UMHealthResearch.org). Participants or their parents gave written informed consent in accordance with the Declaration of Helsinki. All tasks and procedures were approved by the University of Michigan Medical School Institutional Review Board. Participants were paid for their interviews and psychophysiological recordings. Participants were excluded if they made fewer than 10 errors (n = 8), leaving a total of 210 participants. The final sample consisted of 80 males and 130 females of age 8–18 years, with an ethnic/racial breakdown that was 89% Caucasian, 1 % African American, 3% Latino, 3% Asian, and 4% Native American. The case and control groups each had 40 males and 65 females. Female participants were significantly older than male participants (*t*(208) = 3.86, *P*= 0.0002). All participants lived with at least one English-speaking biological parent willing to participate in the research. Tables 1 and 2 summarize the demographic, clinical, behavioral, and ERP data for the participants.

All 105 patients had a lifetime diagnosis of OCD, with 76 having a current diagnosis and 29 a past diagnosis with OCD symptoms that no longer met criteria for diagnosis. Twenty-one patients had a lifetime diagnosis of a tic disorder. Patients were excluded if they had a lifetime diagnosis of autism spectrum disorder, anorexia nervosa, schizophrenia, other psychotic disorder, bipolar disorder, or substance-related disorder. The 105 HC had no history of a specific axis I disorder. Lifetime and current axis I diagnoses were made independently by two clinicians using all sources of information according to *DSM-5* criteria [31]. Participants were excluded if they had a history of intellectual disability, head injury with a loss of consciousness, chronic neurological disorder other than tics, or scores higher than 14 on the Social Communication Questionnaire [32]. Because studies have indicated that treatment with a serotonin reuptake inhibitor (SRI) has no effect on the ERN, 43 patients were enrolled taking a stable dose of an SRI but no other medications [15, 17–19, 27]. Medicated patients were significantly older than unmedicated patients (*t* (103) = 2.72, *P* = 0.008).

### Diagnostic Instruments

All 210 participants were interviewed with the Schedule for Schizophrenia and Affective Disorders for School-Aged Children-Present and Lifetime Version and Schedule for Obsessive-Compulsive and Other Behavioral Syndromes [33, 34]. The maximum and current severity of OCD symptoms in patients was assessed with a modified version of the Children’s Yale-Brown Obsessive Compulsive Disorder Scale [35]. Parents completed the CBCL/6–18 and SCQ about their children [29, 30, 32].

### Stimulus Material and Task Procedures

Participants performed a modified Eriksen flanker task in which arrows appeared on a computer display with congruent (e.g., →→→→→) and incongruent (e.g., →→←→→) conditions [36]. They were instructed to respond by pressing one of two buttons indicating the direction of the central arrow (i.e., right versus left), while ignoring the adjacent arrows, and to respond as quickly and accurately as possible, placing equal emphasis on speed and accuracy (20–22). The flanker task is a test of selective attention and response inhibition that activates the anterior cingulate cortex and pre-supplementary motor cortex [37] and provides a more efficient and reliable measure of ERN amplitude than the Stroop or Go/NoGo tasks [15, 38]. The stimuli remained on the screen for 250 ms, with an interval of 1,500 ms between consecutive trials. Each participant was seated 0.65 meters directly in front of the computer monitor. Following 40 practice trials, each subject completed 8 blocks of 64 trials with the number of completed trials ranging from 256 to 512. Performance feedback was provided after every block to yield an error rate of approximately 10%, with encouragement to focus on speed if there were fewer than four errors or to focus on accuracy if there were more than 10 errors [20–22].

### Electrophysiological Recording and Data Reduction

The electroencephalogram was recorded from DC-104 Hz with 64 Ag/AgCl scalp electrodes, two mastoid electrodes, and two vertical and two horizontal electro-oculogram electrodes, using the BioSemi ActiveTwo system (Amsterdam, the Netherlands). Data were digitized at 512 Hz, referenced to a ground formed from a common mode sense active electrode and driven right leg passive electrode (see http://www.biosemi.com/faq/cms&drl.htm), and rereferenced offline to the average of the two mastoid electrodes. Data were band-pass filtered 0.1–30 Hz using zero-phase shift filters. EEG data were screened using automated algorithms that rejected epochs in which absolute voltage exceeded 500 μV and epochs containing peak to peak activity > 500 μV within 200 ms, with a 100 ms moving window, for midline channels (Fz, FCz, Cz, CPz, Pz). Ocular movement artifacts were then corrected using a regression-based algorithm [39]. After ocular correction, individual trials were rejected if they contained absolute amplitudes >100 μV, a change > 50 μV measured from one data point to the next point, or a maximum voltage difference < 0.5 μV within a trial in any of the midline electrodes.

The mean amplitude of the ERN was computed on error trials in a window from 0 to 80 ms following the incorrect response, relative to a pre-response baseline of −200 to −50 ms. The mean amplitude of the Pe was computed on error trials in a window from 200 to 400 ms following the erroneous response, compared to a pre-response baseline of −200 to −50 ms. The CRN and Pc consisted of the same respective measures computed on correct trials. Amplitudes were calculated for electrodes Fz, FCz, Cz, CPz, and Pz, with the focus of the present study on the ERN measures at Cz and the Pe measures at CPz. Correlational analyses with the ERN and CRN indicate that numerically greater negative values represent higher ERP amplitudes, whereas correlational analyses with the Pe and Pc indicate that numerically greater positive values represent higher ERP amplitudes.

The ΔERN was calculated by subtracting the CRN from the ERN, since it may isolate neural activity unique to error processing from activity more broadly related to response monitoring [7]. The ΔERN is correlated both with the ERN and CRN and is therefore not an independent measure of either ERP [40]. ERN and CRN standardized residual scores (ERN_resid_ and CRN_resid_) were calculated based on measuring the variance leftover in a regression equation in which one score is modeled as a predictor of another score, because they may be preferable to subtraction-based difference scores in separating error processing from response monitoring [22, 40]. Similar measures were calculated for the ΔPe, Pe_resid_, and Pc_resid_.

Behavioral measures included the number of erroneous and correct trials for each subject, as well as accuracy expressed as a percentage of valid trials. Mean reaction times on error and correct trials were calculated separately, and trials were excluded if their reaction times were > 3 standard deviations from the mean. Reaction time and accuracy after errors were evaluated to determine whether there were group differences in post-error behavioral adjustments [7]. Reaction times were analyzed with group as a between-subject factor and response type as a within-subject factor. The mean number of errors per subject contributing to the analysis was 46.8 (SD = 27.2; range = 10–160).

### Statistical Analyses

Student t-tests, χ^2^, analysis of variance, and analysis of covariance tests were used to evaluate group differences in demographic, clinical, and behavioral data. Pearson correlation coefficients were used to examine associations of response-related amplitudes with age, behavioral measures, and clinical measures. Electrocortical indicators (ERN, CRN, ΔERN, ERN_resid_, CRN_resid_, Pe, Pc, ΔPe, Pe_resid_ and Pc_resid_) of performance monitoring were analyzed separately using a repeated-measure analysis of covariance with group (OCD cases and HC) as a between-subject factor, response type (correct and error) as a within-subject factor, and age and accuracy as covariates [7]. Similar analyses were done to compare brain potentials in male and female participants, TR and NTR OCD cases, cases with a current or past OCD diagnosis, and medicated and unmedicated OCD cases. Cohen’s effect size conventions were used to describe the magnitude of effects (small: *d*> 0.20; medium: *d*> 0.50; large: *d*> 0.80) [41].

A multiple linear regression analysis was done with all participants to examine the association of flanker task accuracy with age, ERN, and ΔPe. Separate multiple linear regression analyses were done with all participants to examine the relation of the ERN and ΔPe to age, flanker task accuracy, lifetime OCD diagnosis, and CBCL/6–18 DSM-Oriented Scale scores (obsessive compulsive problems, affective problems, anxiety problems, somatic problems, attention deficit/hyperactivity problems, oppositional defiant problems, and conduct problems) [30, 31]. A multiple regression analysis was done with the OCD cases to examine the association of age at OCD symptom onset with age, lifetime tic diagnosis, ERN, and ΔPe. Analyses were performed with JMP Pro Version 14 software. All tests were two-tailed with α = 0.05.

## Results

### Behavioral Data in Patients with OCD and Healthy Controls

Participants had significantly higher flanker task accuracy on congruent than incongruent trials (paired *t* (209) = 26.42, *P* < 0.0001). OCD cases were significantly less accurate than HC in all trial conditions (all *P* values < 0.02) (Table 1). Overall accuracy was also significantly decreased in both TR OCD and NTR OCD cases compared to HC (both *P* values < 0.05) (Table 2).

Correct responses were significantly slower than incorrect responses (paired *t* (209) = 6.33, *P* < 0.0001). No main effect of group or response type for reaction time and no interaction between group and response type for reaction time reached significance (*P* = 0.63 and *P* = 0.40, respectively). Age had significant positive correlations with accuracy (r = 0.17, *P* = 0.01), post-correct accuracy (*r* = 0.16, *P* = 0.02), and post-error accuracy (*r* = 0.21, *P* = 0.002). Age had significant negative correlations with reaction time on correct (*r* = −0.57, *P* < 0.0001) and incorrect trials (*r* = −0.46, *P* < 0.0001) and a significant positive correlation with post-error slowing (*r* = 0.21, *P* = 0.002).

There were no significant sex differences in flanker task accuracy, reaction time on correct or incorrect trials, or post-error slowing (all *P* values > 0.5). There were no significant differences in accuracy, reaction time on correct or error trials, or post-error slowing between patients with a current and past diagnosis of OCD (all *P* values > 0.09), between medicated and unmedicated patients with OCD (all *P* values > 0.1), or between patients with TR and NTR OCD (all Pvalues > 0.37).

### Event-Related Potential Data in Patients with OCD and Healthy Controls

ERN amplitude was significantly increased (more negative) compared to CRN amplitude (paired *t* (209) = −9.05, *P* < 0.0001), and Pe amplitude was significantly increased (more positive) compared to Pc amplitude (paired *t* (209) = 24.16, *P* < 0.0001). Age in all participants had significant correlations with the ERN (*r* = −0.20, *P* = 0.004), CRN (*r* = 0.24, *P* = 0.0005), and ΔERN (*r* = −0.38, *P* < 0.0001) but not Pe, Pc, or ΔPe (all Pvalues > 0.14). Accuracy had significant correlations with the ERN (*r* = −0.17, *P* = 0.02) but not CRN or ΔERN (both *P* values > 0.07). Accuracy also had significant correlations with the Pe (*r* = 0.18, *P* = 0.009) and ΔPe (*r* = 0.26, *P* = 0.0001) but not Pc (*P* = 0.10). Supplementary Table 1 provides a correlation matrix for age, flanker task accuracy, and ten ERPs.

**Figure 1:**
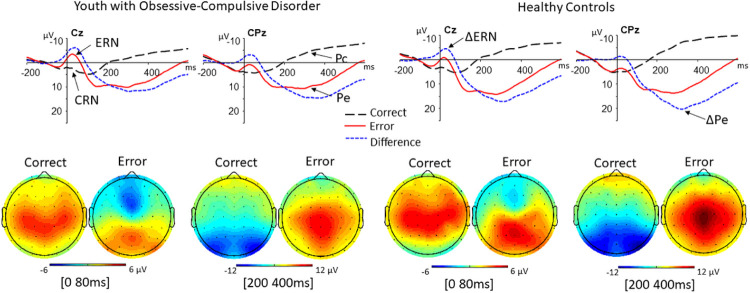
Grand averages of electroencephalogram (EEG) recordings in 105 patients with obsessive-compulsive disorder (AD) and 105 healthy controls (HC). Note: The top images depict response-locked grand average waveforms recorded at the Cz and CPz electrodes for correct and incorrect responses. Responses occurred at 0 ms. The mean amplitude of the error-related negativity (ERN) was computed in a window 0 to 80 ms after incorrect response trials. The mean amplitude of the correct response negativity (CRN) consisted of the same measure computed on correct response trials. The ΔERN was calculated by subtracting the CRN from the ERN. The mean amplitude of the error positivity (Pe) was computed in a window 200 to 400 ms after incorrect response trials. The mean amplitude of the correct positivity (Pc) consisted of the same measure computed on correct response trials. The ΔPe was calculated by subtracting the Pc from the Pe. The bottom images depict the topography of mean amplitudes of erroneous and correct waveforms measured between 0 and 80 ms and between 200 and 400 ms.

ERN amplitude was significantly increased in OCD cases compared to HC (*F*_1, 206_ = 19.36, *P* < 0.0001, Cohen’s *d* = 0.52), with significant effects for accuracy (F_1, 206_ = 7.55, *P* = 0.006) and age *F*_1, 206_ = 6.63, *P* = 0.01) (Table 1; Figure 1). CRN amplitude was significantly enlarged in cases compared to HC (*F*_1, 206_ = 5.58, *P* = 0.02, Cohen’s *d* = 0.24), with significant effects for accuracy (*F*_1, 206_ = 8.73, *P* = 0.004) and age (*F*_1, 206_ = 16.55, *P* < 0.0001). The ΔERN was significantly enhanced in cases compared to HC (*F*_1, 206_ = 6.19, *P* = 0.014, Cohen’s *d* = 0.31), with a significant effect for age (*F*_1, 206_ = 35.16, *P* < 0.0001) but not accuracy (*P* = 0.73).

There was no significant group difference in Pe amplitude (*P* = .14). Pc amplitude was significantly increased in OCD cases compared to HC (*F*_1, 206_ = 4.02, *P* = 0.046, Cohen’s *d* = 0.32), without significant effects for accuracy or age (both *P* values > .08). Moreover, the ΔPe was significantly decreased in cases compared to controls (*F*_1, 206_ = 9.88, *P* = 0.002, Cohen’s *d* = 0.53), with a significant effect for accuracy (F_1, 206_ = 12.76, *P* = .002) but not age (*P* = .07).

Results for the ERN_resid_, CRN_resid_, Pe_resid_, and Pc_resid_ in the two groups are summarized in Table 1. There were no significant sex differences in the ERN, CRN, ΔERN, ERN_resid_, CRN_resid_, Pe, Pc, ΔPe, Pe_resid_, or Pc_resid_ (all *P* values > .05). There were no significant differences in any brain potentials between patients with a current and past OCD diagnosis (all *P* values > .55), between medicated and unmedicated patients with OCD (all *P* values > .10), or between patients with TR OCD and NTR OCD (all *P* values > .35) (Table 2).

### Event-Related Potential Data in Patients with TR OCD, Patients with NTR OCD, and Healthy Controls

In a comparison of the ERN in TR OCD cases, NTR OCD cases, and HC, there were significant effects for group (*F*_2, 205_ = 9.70, *P* < .0001), age (*F*_1, 205_ = 6.72, *P* = .01), and accuracy (*F*_1, 205_ = 7.52, *P=* .007) (Table 2). ERN amplitude was significantly increased in TR OCD cases compared to HC (*F*_1, 122_ = 6.09, *P* = .02, Cohen’s *d* = .47), with no significant effects for age or accuracy (both *P* values > .07). ERN amplitude was also significantly enlarged in NTR OCD cases compared to HC (*F*_1, 185_ = 16.72, *P* < .0001, Cohen’s *d* = .53), with significant effects for age (*F*_1, 185_ = 8.44, *P* = .004) and accuracy (*F*_1, 185_ = 7.65, *P* = .006). In a comparison of the ΔERN in the three groups, there were significant effects for group (*F*_2, 205_ = 3.69, *P* < .03) and age (*F*_1, 205_ = 36.36, *P* < .0001) but not accuracy (*P* = .74). The ΔERN was significantly increased in TR OCD cases compared to HC (*F*_1, 122_ = 4.46, *P* = .04, Cohen’s *d*= .32), with a significant effect for age (*F*_1, 122_ = 25.97, *P* < .0001) but not accuracy (*P* = .32). The ΔERN was also significantly augmented in NTR OCD cases compared to HC (*F*_1, 185_ = 3.97, *P* = .048, Cohen’s *d* = .31), with a significant effect for age (*F*_1, 185_ = 37.04, *P* < .0001) but not accuracy (*P* = .77).

In a comparison of the ΔPe in TR OCD cases, NTR OCD cases, and HC, there were significant effects for group (*F*_2, 205_ = 4.99, *P* = .008) and accuracy (*F*_2, 205_ = 12.72, *P* = .0005) but not age (*P* = .06) (Table 2; Figure 3). The ΔPe was significantly decreased in TR OCD cases compared to HC (*F*_1, 122_ = 4.43, *P* = .04, Cohen’s *d* = .57), with significant effects for accuracy (*F*_1, 122_ = 8.02, *P* = .005) and age (*F*_1, 122_ = 4.14, *P* = .04). The ΔPe was also significantly diminished in NTR OCD cases compared to HC (*F*_1, 185_ = 7.73, *P* = .006, Cohen’s *d* = .52), with a signi**fi**cant effect for accuracy (*F*_1, 185_ = 13.80, *P* = .0003) but not age (*P* = .77). Results for the ERN_resid_, CRN_resid_, Pe_resid_, and Pc_resid_ in the three groups are summarized in Table 2.

### Flanker Task Accuracy and Event-Related Potential Data in Patients with OCD and Healthy Controls

Because of the group differences in the ERN and ΔPe noted above along with the strong correlation of accuracy with age, a multiple linear regression analysis was done using age and both brain potentials as predictors to determine their associations with flanker task accuracy as the dependent variable. Age and both brain potentials were significantly associated with flanker task accuracy in all participants, with a more negative ERN and more positive ΔPe associated with higher accuracy (Table 3).

### Clinical and Event-Related Potential Data in Patients with OCD and HC

Separate multiple linear regression analyses were done with all participants with either the ERN or ΔPe as the dependent variable and age, flanker task accuracy, lifetime OCD diagnosis, and CBCL/6–18 *DSM*-Oriented Scale scores as predictors. The ERN had significant associations with CBCL/6–18 Anxiety Problems Scale scores, age, and accuracy in the full model (Table 4). Backward stepwise regression analysis determined that only Anxiety Problems Scale scores and age were significantly associated with the ERN in the reduced model. The ERN had a significant negative correlation with Anxiety Problems Scale scores in OCD cases (*r* = −0.30, *P* = 0.002) but not HC (*P* = 0.46).

The ΔPe had significant associations with accuracy and age in the full model (Table 5). Backward stepwise regression analysis found that accuracy, CBCL/6–18 Obsessive Compulsive Problems Scale scores, and age were significantly associated with the ΔPe in the reduced model. The ΔPe had no significant correlations with Obsessive Compulsive Problems Scale scores in either OCD cases or HC alone (both *P* values > 0.18). Supplementary Table 2 provides a correlation matrix for ten brain potentials and seven CBCL/6–18 DSM-Oriented Scales.

### Age at Onset of OCD Symptoms and Event-Related Potential Data in Patients with OCD

A multiple linear regression analysis was done with the OCD cases to examine the relation of age, history of tic disorder, ERN, and ΔPe to age at OCD symptom onset. Age at OCD symptom onset had significant associations with age and history of tic disorder in the full model (Table 6). Backward stepwise regression analysis found that tic disorder, age, and ΔPe with significantly associated with age at OCD symptom onset in the reduced model. The ΔPe had a significant positive correlation with age at OCD symptom onset in NTR OCD cases (*r* = 0.25, *P* = 0.02) but not TR OCD cases (*P* = 0.62). ERN amplitude had no significant correlations with age at OCD symptom onset in either TR OCD or NTR OCD cases (both *P* values > 0.19).

## Discussion

Consistent with previous reports of altered neural correlates of performance monitoring in OCD, we found an increased ERN, ΔERN, and ERN_resid_ in a large sample of children and adolescents with a history of OCD (Table 1) [7, 8, 15, 17–19]. The moderate effect size (Cohen’s *d* = 0.52) for the enlarged ERN is comparable to that reported in a meta-analysis of performance monitoring studies in adults and children with OCD (Hedge’s *g* = 0.54) [8]. Our finding of an increased CRN in pediatric OCD cases is consistent with several studies of adult OCD cases [18, 19, 27]. We also found an increased ERN, ΔERN, and ERN_resid_ in both TR and NTR OCD cases (Table 2), with similar effect sizes for the two OCD subtypes (Cohen’s *d* = 47 and 0.53, respectively). Although an increased ERN has been found in a meta-analysis of studies examining error-related brain activity in patients with either OCD or Tourette’s disorder, there are no previous studies demonstrating an increased ERN in both TR and NTR OCD cases [8]. The results support the hypothesis that an enlarged ERN may serve as a transdiagnostic biomarker for OCD and tic disorders as well as some anxiety disorders [8, 17–22].

In the multiple regression analysis with the ERN as the dependent variable in the total sample, only CBCL/6–18 Anxiety Problems Scale scores and age were significant predictors in the reduced model (Table 4 and Supplementary Table 2). The ERN had a significant correlation with Anxiety Problems Scale scores in OCD cases but not HC. The results suggest the RDoC sustained threat construct may be useful in understanding the pathophysiology of OCD, in which the ERN may specifically reflect sensitivity to the degree in which errors are evaluated as threatening (15, 42).

In contrast to most studies of performance monitoring in OCD, we found a *decreased* ΔPe and Pe_resid_ along with an *increased* Pc and Pc_resid_, in pediatric OCD cases (Table 1) [8, 26, 27]. Youth with either TR or NTR OCD had similar reductions in the ΔPe (Table 2). Our ΔPe finding requires replication in other large pediatric OCD samples. Studies examining the ERN and ΔPe in other psychiatric disorders will be necessary to determine whether the combination of an increased ERN and a decreased ΔPe is specific to pediatric OCD.

In the multiple regression analysis with the ΔPe as the dependent variable in the total sample, flanker task accuracy, CBCL/6–18 Obsessive Compulsive Problems Scale scores, and age were significant predictors in the reduced model (Tables 5 and Supplementary Tables 1 and 2). In the multiple regression analysis with age at OCD symptom onset as the dependent variable in OCD cases alone, the association between the ΔPe and symptom onset age suggests atypical development of the Pe or Pc may be involved in the pathogenesis of pediatric OCD (Table 6). However, the correlation between the ΔPe and age at OCD symptom onset was significant in the NTR OCD group but not the smaller TR OCD group. The associations of the ΔPe with the Obsessive Compulsive Problems Scale scores and age at OCD symptom onset indicate that the ΔPe may be more directly involved in the pathogenesis of OCD symptoms than the ERN and that interventions augmenting the ΔPe may diminish OCD symptoms.

In contrast to studies finding normal or increased flanker task accuracy in adults with OCD, we found *decreased* flanker task accuracy in youth with OCD, including impairment on congruent and incongruent trials and after correct and incorrect trials (Table 1) [18–20]. TR and NTR OCD cases had similar impairments in accuracy (Table 2). OCD cases had decreased flanker task accuracy despite having an increased ERN, which correlated with higher task accuracy in all participants, indicating that an enlarged ERN may develop over time in youth with OCD to compensate for deficits in error monitoring [42] (Table 3). The association between a reduced ΔPe and flanker task accuracy suggests that, as the Pc and Pe become closer in amplitude, error signaling may become compromised and cause uncertainty about the correctness or adequacy of a response: that is, the reduced ΔPe in pediatric OCD may reflect a defect in the post-decisional evidence accumulation process that impairs decision accuracy, decision confidence, and subsequent behavioral adjustments [11, 23, 24].

Our study has limitations requiring further consideration. Participants were primarily Caucasian and treatment was uncontrolled; however, it is doubtful that brain potentials would be different with a more diverse or untreated sample [8, 15–17, 25, 27, 42]. Children younger than 8 years were not enrolled. Age at OCD symptom onset was assessed retrospectively rather than prospectively. Many of the findings are correlational, requiring experimental studies to establish any causal relationships between the variables.

## Summary

The ERN and Pe are components of the ERP following an error that are potential mechanistic biomarkers for OCD [5, 7–11, 15, 17–21]. The study examined the ERN, Pe, and flanker task accuracy in 105 OCD cases and 105 matched healthy controls (HC), ages 8 to 18 years, with 21 cases having a tic disorder history. Compared to HC, the ERN was increased in OCD cases and in TR and NTR OCD cases. The results support the hypotheses that an enlarged ERN may serve as a transdiagnostic biomarker for OCD and tic disorders as well as some anxiety disorders [8, 17–22]. Compared to HC, the ΔPe was *decreased* in OCD cases and in TR and NTR OCD cases. A lower ΔPe in OCD cases was associated with an earlier age at OCD symptom onset, suggesting that atypical development of the Pc or Pe may be involved in the pathogenesis of pediatric OCD. Compared to HC, flanker task accuracy was *decreased* in OCD and in TR and NTR OCD cases, whereas higher flanker task accuracy was associated with increased ERN and ΔPe measures in the total sample. The association between a reduced ΔPe and flanker task accuracy may reflect a defect in the post-decisional evidence accumulation process that, as the Pc and Pe become closer in amplitude, impairs decision accuracy, decision confidence, and subsequent behavioral adjustments [11, 23, 24]. The associations of the ΔPe with OCD symptom onset age and flanker task accuracy in pediatric OCD cases suggests that interventions augmenting the ΔPe may diminish OCD symptoms.

## Data Availability

Data and materials are available upon request to the corresponding author.
